# The transcription factor Foxd3 induces spinal cord ischemia-reperfusion injury by potentiating microRNA-214-dependent inhibition of Kcnk2

**DOI:** 10.1038/s12276-019-0370-8

**Published:** 2020-01-21

**Authors:** Ran Li, Kunchi Zhao, Qing Ruan, Chunyang Meng, Fei Yin

**Affiliations:** Department of Spine Surgery, China-Japan Union Hospital, Jilin University, Changchun, 130033 P.R. China

**Keywords:** Genetics, Molecular biology

## Abstract

Spinal cord injury after surgical repair of the thoracic or thoracoabdominal aorta is a devastating complication that is associated with pathological changes, including inflammation, edema, and nerve cell damage. Recently, microRNA (miRNA)-modulated control of spinal cord injury has been actively investigated. This study aims to clarify the regulatory effect of miR-214-mediated inhibition of Kcnk2 following spinal cord ischemia-reperfusion injury (SCII) and the possible underlying mechanisms. SCII was induced in rats by occluding the aortic arch followed by reperfusion. Gain-of-function and loss-of-function experiments were conducted to explore the modulatory effects of Foxd3, miR-214 and Kcnk2 on PC12 cells under hypoxia/reoxygenation (H/R) conditions. MiR-214 and Kcnk2 were poorly expressed, while Foxd3 was highly expressed in the rat spinal cord tissues and H/R-treated PC12 cells. Kcnk2 overexpression enhanced the viability and inhibited the apoptosis of the H/R-treated PC12 cells. Notably, Foxd3 activated miR-214, and miR-214 targeted Kcnk2. In addition, upregulation of Kcnk2 or knockdown of Foxd3 promoted the cell viability and reduced the apoptosis of the H/R-treated PC12 cells. Overall, our study identified a novel mechanism of Foxd3/miR-214/Kcnk2 involving SCII, suggesting that either Foxd3 or miR-214 may be a novel target for the treatment of SCII.

## Neurology: Seeking salvation for spines after surgery

Therapeutic strategies that protect neurological function could prevent serious side effects associated with surgery of the aorta. Patients undergoing aortic surgery for aneurysm or other conditions are at risk of spinal cord damage associated with reduced blood flow during surgery and the subsequent restoration of normal circulation. Researchers led by Fei Yin at Jilin University in Changchun, China, have identified molecular mechanisms that can potentially increase this neurological harm. They show that the combination of low oxygen and reperfusion triggers production of a protein called Foxd3, which switches off cellular pathways that otherwise protect and repair vulnerable tissues. By interfering with this Foxd3-mediated response, Yin’s team was able to protect motor function and preserve the survival of spinal cord neurons in a rat model of this condition, suggesting a potential therapeutic avenue for heart surgery patients.

## Introduction

Spinal cord ischemia-reperfusion injury (SCII) is a serious complication that commonly occurs following thoracoabdominal aortic surgery and results in severe neurological deficits^1^. There are two main types of spinal cord ischemia: infarction of the radicular arteries and extensive spinal cord hypoperfusion^2^. SCII during surgeries of the thoracoabdominal vasculature could result in severe complications, such as paraplegia^3^. MicroRNAs (miRNAs), which preferentially restrain the translation of many cellular proteins, have been reported to induce proinflammatory responses and regulate neuroinflammation in various pathologies, including ischemic stroke and spinal cord injury (SCI)^4^. MiRNAs may be involved in physical activity-related attenuation of the vascular risk and remodeling in individuals with SCI^5^. These findings have resulted in increased interest in the potential regulatory roles of miRNAs in SCII and have prompted further investigations.

MiRNAs play an important role in cell functions in most biological processes. Typical miRNAs negatively regulate the expression of multiple target genes^6^. Abnormalities in miRNAs may result in abnormal conditions such as microglial hyperactivation, persistent neuroinflammation and abnormal polarization of brain macrophages, which may contribute to the pathogenesis of neurological diseases such as glioma, ischemia and SCI^7^. Moreover, miRNAs can regulate the pathophysiological process of SCI and may be diagnostic and prognostic biomarkers for this condition^8^. MiR-214 was found to be overexpressed in ulcerative colitis tissues^9^ and human melanomas^10^. Moreover, miR-214 could modulate ischemia-reperfusion (I/R)-induced myocardial injury^11^. The modular synergy between miRNAs and neuron subtype-specific transcription factors has been demonstrated to promote system-specific neuron reprogramming, providing a universal platform for the efficient generation of human neurons with different subtypes^12^. In signal transduction networks, miRNAs could directly or indirectly affect the expression and secretion of proinflammatory proteins through transcription factors and other regulatory factors^13^. Furthermore, miR-214 was found to be downregulated in colorectal cancer, and the transcription factor forkhead box D3 (Foxd3) was positively correlated with miR-214^14^. In addition, Foxd3 could maintain multipotent neural crest stem cells by suppressing non-neural differentiation^15^. This molecule also maintains the pluripotency of three distinct divergent progenitor populations in early mammalian embryos^16^. The mammalian K2P2.1 potassium channel (Trek-1, Kcnk2) is a two-pore-domain potassium channel that participates in regulating the resting membrane potential of neurons^17^. Kcnk2 was highly expressed in neural stem cells^18^. Therefore, we hypothesized that Foxd3 could modulate miR-214-dependent Kcnk2 expression and might contribute to the exacerbation of SCII.

## Materials and methods

### Ethics statement

The animal experiments in this study were conducted according to the guidelines of animal care and use, and were approved by the Animal Ethics Committee of the Center of Laboratory Animals, Jilin University.

### Establishment of a SCII rat model

Twenty male Sprague-Dawley (SD) rats (aged 8-weeks-old) were purchased from the Center of Laboratory Animals, Jilin University, China (license no. SCXK(Ji)2008-0005) and were then divided into the experimental group and the sham group, with 10 rats in each group. A model of SCII was prepared as previously described^19^. The rats were intraperitoneally injected with 10% chloral hydrate (3 mL/kg) and were fixed on their side. A 5-cm incision was made down from the lower edge midline of the left ribs. The left kidney was then located, followed by the abdominal aorta along the renal artery, which was ligated with a 10-g bulldog clamp below the renal artery for 1 h. The bulldog clamp was then removed, and the abdominal cavity was closed after it was washed with penicillin. The rats in the sham surgery group only received a laparotomy without ligation of the abdominal aorta. The model was deemed to be successfully established if neurological deficits appeared in the hindlimb. The controls were not given any treatment. The rats were euthanized after the experiment, and the spinal cord (L2-5) tissues were collected for subsequent analyses.

### Lentivirus delivery

Lentivirus (Lv)-negative control (NC), Lv-Kcnk2, and Lv-sh-Foxd3 were purchased from Shanghai Hanbio (Shanghai, China). A laminectomy was performed at the thoracic level under pentobarbital anesthesia. A polyethylene catheter (inner diameter 0.28 mm and outer diameter 0.61 mm, PE10, Portex, Smith Medical, Kent, UK) was passed through the T9-12 tail, and the 2-cm free end was exposed to the upper thoracic region. The Kcnk2-containing lentivirus (Lv-Kcnk2) and its control (Lv-NC) were both prepared and titered to 1 × 10^9^ transfection units/mL. All 10 rats in each group were intrathecally injected with 100 μL of lentivirus. After five days, the descending aorta was cross-clamped for 14 min to induce spinal cord ischemia, and the hind limb motor function was evaluated after 2 days.

### Reverse transcription quantitative polymerase chain reaction (RT-qPCR)

Total RNA was extracted using an RNeasy Mini Kit (Qiagen, Valencia, CA, USA). Subsequently, the extracted RNA was reverse transcribed into complementary DNA (cDNA) using a reverse transcription kit (RR047A, TaKaRa Bio Inc., Otsu, Japan). Polyadenylation was detected on the separated RNA using the tailing method with an NCode™ miRNA First-Strand cDNA Synthesis Kit (MIRC10, Invitrogen, Carlsbad, CA, USA). RT-qPCR was performed using a SYBR Premix EX Taq kit (RR420A, TaKaRa Bio, Inc., Otsu, Japan) on a quantitative PCR instrument (ABI 7500, Applied Biosystems, Carlsbad, CA, USA). General primers provided by the NCode™ miRNA First-Strand cDNA Synthesis Kit were regarded as miRNA negative primers, and other primers were synthesized by Shanghai Sangon Biotech (Shanghai, China) (primer sequences are shown in Table 1). U6 and glyceraldehyde-3-phosphate dehydrogenase (GAPDH) were used as controls. The relative expression of the genes was calculated through relative quantification (2^−^△△^Ct^ method).

**Table 1:** Primer sequences for RT-qPCR.

Target	Primer sequences
FOXD3	F: 5′-CGAGCAAGCCCAAGAAC-3′
	R: 5′-TGCTGATGAACTCGCAGAT-3′
Kcnk2	F: 5′-GTGGAGGACACATTTATTAAGT-3′
	R: 5′-GAAGAGGACACAGCCAAACA-3′
miR-214	F: 5′-AGCCGACAGCAGGCACAGACA-3′
	R: 5′-TGGTGTCGTGGAGTCG-3′
Bax	F: 5′-TGGTTGCCCTCTTCTACTTTG-3′
	R: 5′-GTCACTGTCTGCCATGTGGG-3′
GAPDH	F: 5′-ACTCCCATTCTTCCACCTTTG-3′
	R: 5′-CCCTGTTGCTGTAGCCATATT-3′
U6	F: 5′-GTGCTCGCTTCGGCAGCACATATAC-3′
	R: 5′-AAAAATATGGAACGCTCACGAATTTG-3′
Ki67	F: 5′-ATTTCAGTTCCGCCAATCC-3′
	R: 5′-GGCTTCCGTCTTCATACCTAAA-3′

*RT-qPCR* reverse transcription-quantitative polymerase chain reaction, *F* forward, *R* reverse, *FOXD3* forkhead box D3, *Kcnk2* potassium two-pore domain channel subfamily K member 2, *miR-214* microRNA-214, *Bax* BCL-2 associated X, *GAPDH* glyceraldehyde-3-phosphate dehydrogenase

### Western blot analysis

The tissues or cells were incubated in an ice bath with radioimmunoprecipitation assay lysis buffer containing phenylmethylsulfonyl fluoride at 4 °C for 30 min, after which centrifugation was carried out at 8000 × *g* for 10 min to extract the total protein. The total protein concentration was detected by a bicinchoninic acid protein assay kit. The samples were then separated by sodium dodecyl sulfate-polyacrylamide gel electrophoresis and transferred onto a polyvinylidene fluoride membrane. Subsequently, the membrane was blocked in 5% skim milk at room temperature for 1 h, followed by incubation overnight with diluted primary rabbit anti-rat antibodies against Foxd3 (ab67758, 1: 1000), Kcnk2 (TREK-1, ab90855, 1: 2000), Bax (ab32503, 1: 1000), Ki67 (ab16667, 1: 1000), and GAPDH (ab181603, 1: 10000) (Abcam, Inc., Cambridge, UK). Then, the membrane was probed with horseradish peroxidase-labeled goat anti-rabbit antibodies against immunoglobulin G (IgG) H&L (ab97051, 1: 2000, Abcam, Inc., Cambridge, UK) for 1 h. Finally, the protein bands were visualized using the enhanced chemiluminescence Fluorescence Detection Kit (BB-3501, GE Healthcare, Little Chalfont, Buckinghamshire, UK) under dark conditions, followed by exposure and photography using a Bio-Rad Image Analysis System (Bio-Rad, Hercules, CA, USA). The scanned images were quantitated using Quantity One v4.6.2 software, with GAPDH as an internal reference.

### Cell culture and treatment

The PC12 nerve cell line was cultured in a 37 °C incubator with 5% CO_2_ in Dulbecco’s modified Eagle’s medium (DMEM, Gibco, Carlsbad, CA, USA) containing 10% fetal bovine serum, 100 U/mL penicillin and 100 μg/mL streptomycin (HyClone, GE Healthcare, Little Chalfont, Buckinghamshire, UK). PC12 cells were then maintained in DMEM (Gibco, Carlsbad, CA, USA) without glucose and placed in a hypoxic chamber of a Ruskin Bugbox Plus (Ruskinn Technology, Ltd., Cardiff, UK) at 37 °C with 95% N_2_ and 5% CO_2_ for 2 h. Following the development of hypoxia, the cells were cultured in a 37 °C incubator with 95% air and 5% CO_2_ for 12 h, and the oxygen-glucose deprivation medium was renewed with normal DMEM. The control cells were cultured under normal conditions.

pcDNA3.1 was used to establish the overexpression (oe-) plasmids. Cell density was adjusted according to the cell growth, and then, the cells were seeded into 6-well plates. When the cell density reached ~80–90% confluence, Lipofectamine 2000 (Invitrogen, Inc., Carlsbad, CA, USA) was used for the cell transfection. Then, the cells were transfected with the mimic NC, miR-214 mimic, pcDNA3.1 (oe-NC), pcDNA3.1-Kcnk2 (oe-Kcnk2), short hairpin (sh)-NC and sh-Foxd3 plasmids. All of the above plasmids were supplied by Shanghai GenePharma Co., Ltd. (Shanghai, China). The dosages used for mimic NC and miR-214 mimic were 50 nM^20,21^, and 20 nM was used for pcDNA3.1, pcDNA3.1-Kcnk2, sh-NC and sh-Foxd3^22^. After 24 h of transfection, the cells underwent hypoxia (2 h) and reoxygenation (22 h) and were the collected for subsequent experiments.

### Dual luciferase reporter gene assay

The sequences with the predicted binding sites between miR-214 and the 3′-untranslated region (3′-UTR) of Kcnk2 were inserted into the gene vector pmirGLO (Promega Corp., Madison, WI, USA). Next, wild type-Kcnk2-3′UTR (WT-Kcnk2-3′-UTR) and mutant-Kcnk2-3′-UTR (MUT-Kcnk2-3′-UTR) were synthesized by Shanghai GeneChem Co., Ltd. (Shanghai, China). The WT-Kcnk2-3′-UTR and MUT-Kcnk2-3′-UTR constructs were then cotransfected with NC mimic or miR-214 mimic into PC12 cells. After a 24-h transfection, the cells underwent 2 h of hypoxia and 22 h of reoxygenation and were collected and lysed. According to the manufacturer’s instructions of the Dual Luciferase Detection Reagent Kit (K801-200, BioVision, Milpitas, CA, USA), the luciferase reporter gene was detected using a Dual-Luciferase Reporter Gene Analysis System (Promega Corp., Madison, WI, USA). The luciferase activity of the target reporter gene was determined according to the ratio of the relative light units (RLU) of firefly luciferase divided by the RLU of Renilla luciferase, with Renilla luciferase as an internal control.

The three most likely binding sites between Foxd3 and the miR-214 promoter were determined by the UCSC website (http://genome.ucsc.edu/) and the JASPAR website (http://jaspar.genereg.net/). Truncated or mutated binding sites of the recombinant luciferase reporter gene vectors were constructed, followed by cotransfection with the Foxd3 expression vector into the PC12 cells. The Dual-Luciferase Reporter Gene Assay was used to verify the specific binding sites of Foxd3 and the miR-214 promoter.

### Chromatin immunoprecipitation (ChIP)

The PC12 cells were fixed with formaldehyde for 10 min to produce DNA-protein crosslinks. The ultrasonic breaker was set to 10 s per ultrasonic cycle with 10-s intervals for 15 cycles to disrupt the chromatin (200–1000 bp). Then, the supernatant was collected after a 12,000 g centrifugation for 10 min at 4 °C and divided into two tubes. The chromatin fragments were incubated with rat antibody to IgG (5873 S, 1: 20, Cell Signaling Technology, Danvers, MA, USA) or Foxd3 antibody (5337 S, 1: 200, Cell Signaling Technology, Danvers, MA, USA) at 4 °C overnight, with IgG as the NC. The proteins or DNA that could bind to Foxd3 were sedimented by centrifugation using Pierce protein A/G Magnetic Beads (88803, Thermo Fisher Scientific, San Jose, CA, USA). The sediment was centrifuged at 12000 × *g* for 5 min. The nonspecific complex was washed away from the precipitate, the crosslinking was reversed at 65 °C overnight, and the DNA fragments were purified and recovered by phenol/chloroform extraction. The primers (forward: 5′-TGATTCAGATTTTCACTTGGGGTATG-3′, reverse: 5′-AATTAAACATTAAACATTATGGAACT-3′) were designed to amplify a 691 bp amplification product containing the Foxd3 binding site with the miR-214 DNA promoter sequences of site 2 (1879–1890 bp) and a 111 bp product from the transcription start site. The distal primers that amplified the sequence distant from the miR-214 promoter region were designed as an NC for site 2 (forward: 5′-ACTCTCCAGCCCAGCCCTCCCCCTTT-3′, reverse: 5′-TATTTCTGGTGTTTCTATTA-3′). The length of the distal primer amplification product was 326 bp, and the distance from the transcription start site (TSS) is 4735 bp. The recovered and purified DNA fragments were used as the amplification template, while site 2 primer and distal primer (NC) were added to verify whether site 2 of miR-214 is the binding site of the transcription factor Foxd3 by RT-qPCR.

### Cell viability assay

Rat spinal cord cells were cultured overnight with 100 μL of culture medium in 96-well plates in a 37 °C incubator with 5% CO_2_, with the blank wells set. After 24 h of transfection, hypoxia (2 h) and reoxygenation (22 h) were performed. Subsequently, the cells in each well were reacted with 10 μL of Cell Counting Kit-8 (CCK-8) solution (Dojindo Laboratories, Kumamoto, Japan) at 37 °C for 1–2 h. The optical density (OD) value was detected at 450 nm in each well with a microplate reader.

### Basso, Beattie, and Bresnahan **scoring**

The motor function of the hind limbs was assessed 7 days after surgery using the Basso, Beattie, and Bresnahan (BBB) motor rating scale, based on the motor capacity of the SCII rats. The BBB score ranged from 0 to 21, with 0 indicating no locomotion and 21 indicating normal motor function^23,24^. The score was determined by two independent observers who had not performed the experiment, and the scores of the two observers were averaged.

### Terminal deoxynucleotidyl transferase dUTP nick end labeling staining

Cell apoptosis was measured by Terminal deoxynucleotidyl transferase dUTP nick end labeling (TUNEL) assays (Roche Molecular Systems, Inc., Branchburg, NJ, USA) according to the manufacturer’s protocol. Briefly, the spinal cord tissues or PC12 cells were fixed with 4% formaldehyde for 20 min, permeabilized in the permeabilization solution for 10 min at room temperature, and finally labeled with TUNEL at 37 °C in a humidified environment for 60 min. After TUNEL staining, the sections underwent incubation with 6-diamidino-2-phenylindole solution for 5 min to stain the nuclei. Finally, five fields were randomly selected in each section, and the average number of apoptotic cells per 200 cells was determined.

### Statistical analysis

Statistical analyses were performed using SPSS 21.0 statistical software (IBM, Armonk, N.Y., USA). Measurement data are presented as the mean ± standard deviation. An unpaired *t*-test was used for comparison of unpaired data showing homogeneity of variance and a normal distribution. Data from multiple groups were analyzed by one-way analysis of variance (ANOVA), followed by Tukey’s post hoc test. Data from multiple groups at different time points were compared by repeated measures ANOVA, followed by a Bonferroni post hoc test. The correlation between two indicators was evaluated by Pearson correlation analysis. A value of *p* < 0.05 was considered statistically significant.

## Results

### Upregulation of Kcnk2 could alleviate SCII

**Fig. 1:**
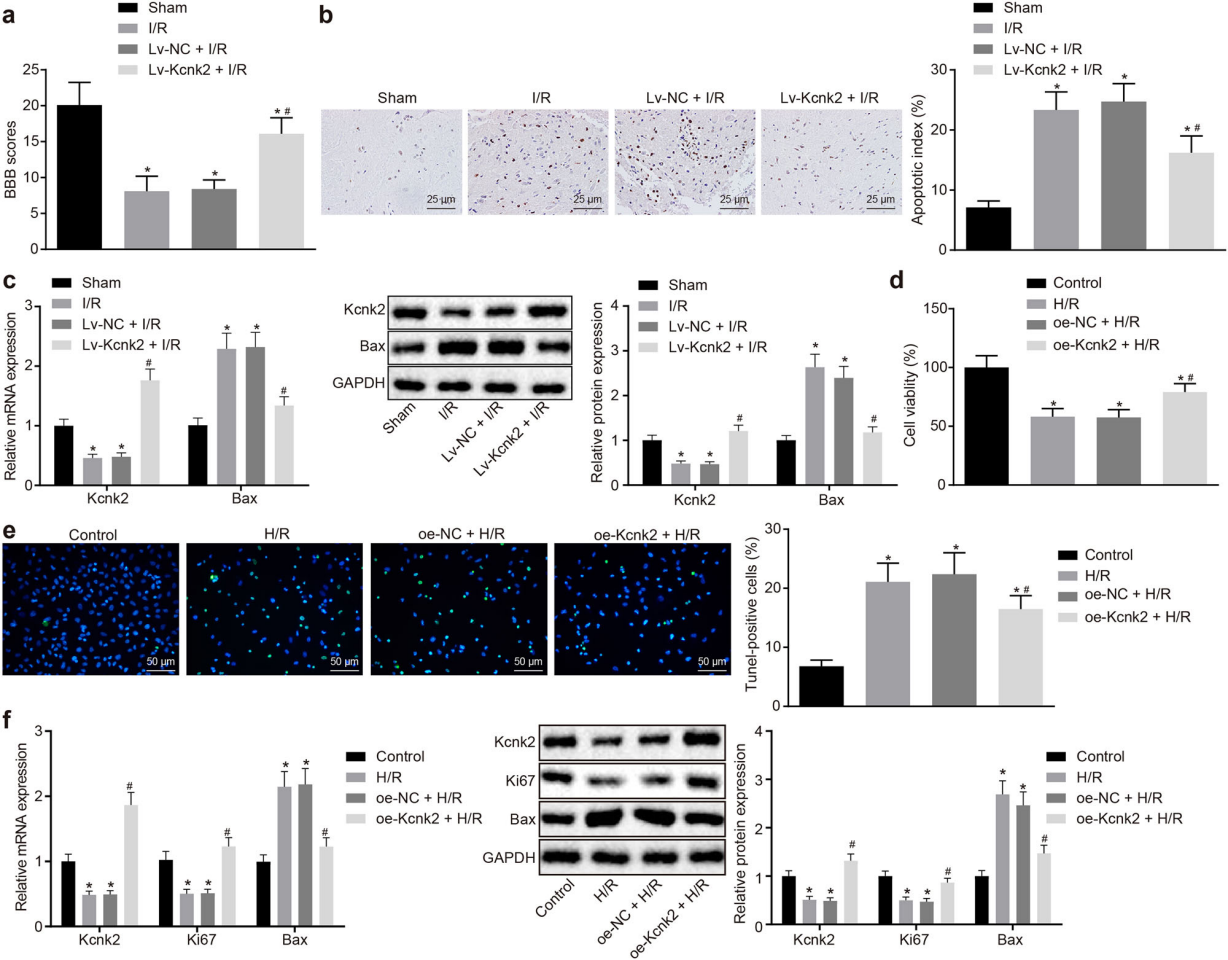
High expression of Kcnk2 may alleviate the progression of SCII. **a** The locomotor function of the hind limbs was evaluated by the BBB locomotor rating scale. *n* = 10. **b** The apoptosis of spinal neurons in the spinal cord tissues of rats was detected by TUNEL staining (×400). **c** The expression of Kcnk2 and Bax in the spinal cord tissues of rats was identified by RT-qPCR and western blot analysis. **d** The cell viability of each group was assessed by CCK-8 assays. **e** Cell apoptosis was tested by TUNEL staining (×200). **f** The expression of Kcnk2, Ki67 and Bax was determined by RT-qPCR and western blot analysis. *p < 0.05 *vs*. the sham group or the control group. #*p* < 0.05 vs. the I/R group or the Lv-NC + I/R group or the H/R group or the oe-NC + H/R group. Statistical values were measured and are expressed as the mean ± standard deviation. One-way ANOVA was used for the comparisons of data from multiple groups, followed by Tukey’s post hoc test.

Based on previous literature, Kcnk2 (TREK-1) plays an important role in the neuroprotection of spinal cord ischemia^25^. The sham-operated rats were regarded as the control group, while the I/R rats were established by occluding the aortic arch and were then infected with negative control lentivirus (Lv-NC + I/R) or Kcnk2-containing lentivirus (Lv-Kcnk2 + I/R) or were not injected (I/R). Next, the hind limb motor function of the rats was evaluated using the BBB locomotor rating scale after 7 days of surgery. The results revealed that I/R exposure resulted in significantly reduced BBB scores in the experimental rats compared with the sham-operated rats (*p* *<* 0.05). Injection of Lv-NC did not have any effect on the BBB score; however, Lv-Kcnk2 injection after exposure to I/R significantly enhanced the BBB score to a similar level as the sham controls (*p* *<* 0.05; Fig. 1a). Therefore, we inferred that overexpression of Kcnk2 could improve the motor function of the hind limb in the I/R rats. Furthermore, apoptosis of rat spinal neurons was detected by TUNEL staining, and the expression of Kcnk2 and Bax was determined by western blot analysis. The results revealed that the apoptosis rate of the spinal neurons and the expression of Bax were strongly elevated, whereas the Kcnk2 expression was diminished in the rats exposed to I/R compared to the sham-operated rats (*p* < 0.05). However, Lv-Kcnk2 injection significantly reduced the apoptosis rate and Bax expression induced by the I/R procedure and restored Kcnk2 expression in the spinal neurons of the rats (*p* *<* 0.05; Fig. 1b, c).

The roles of Kcnk2 in the responses to I/R injury in vitro were investigated by first exposing the PC12 cells to H/R (H/R), followed by transfection with plasmids overexpressing the negative control (oe-NC + H/R) and overexpressing Kcnk2 (oe-Kcnk2 + H/R), with PC12 cells without H/R exposure as a control. The evaluation of cell viability (Fig. 1d), apoptosis (Fig. 1e) and the expression of Kcnk2, Bax and Ki67 (Fig. 1f) revealed that the H/R treatment significantly reduced the cell viability and Kcnk2 and Ki67 protein expression, while promoting apoptosis and the expression of Bax in PC12 cells compared to control cells (*p* *<* 0.05). In contrast, the cell viability and Kcnk2 and Ki67 protein expression were substantially enhanced, whereas apoptosis and the expression of Bax were inhibited in the PC12 cells transfected with oe-Kcnk2 following H/R exposure (*p* *<* 0.05). The aforementioned results indicated that the overexpression of Kcnk2 alleviated SCII by improving the motor functions and suppressing the apoptosis induced by I/R.

### MiR-214 targets and negatively regulates Kcnk2

**Fig. 2:**
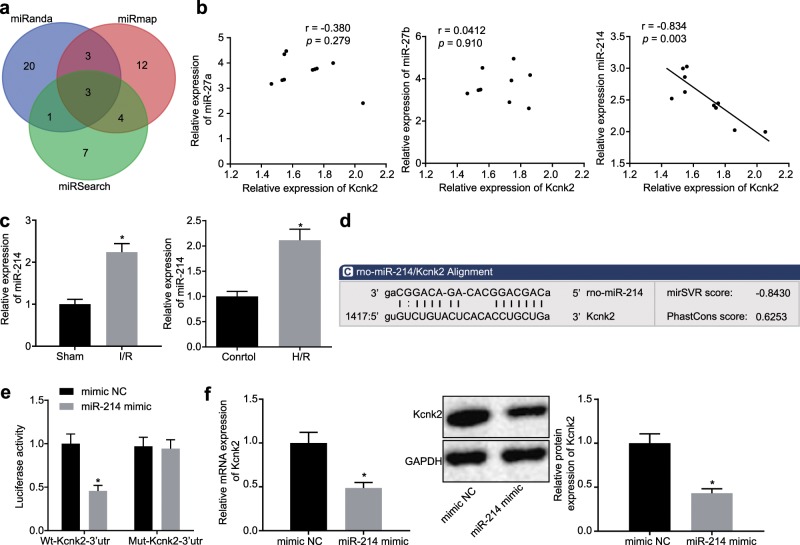
MiR-214 negatively regulates Kcnk2. **a** Prediction of the miRNAs targeting Kcnk2 was performed using the miRanda, miRSearch and miRmap databases. **b** The correlations of the miR-27a, miR-27b, miR-214, and Kcnk2 expression levels in the rat spinal cord tissues following I/R were analyzed by Pearson correlation analysis. **c** The expression levels of miR-214 in the rat spinal cord tissues following I/R and the PC12 cells exposed to H/R were detected by RT-qPCR. *p < 0.05 *vs*. the sham group or the control group. **d** The predicted binding sites between miR-214 and Kcnk2 are shown. **e** The targeting relationship between miR-214 and Kcnk2 was measured by dual luciferase reporter gene assays. *p < 0.05 vs. the mimic NC group**. f** Kcnk2 expression in PC12 cells was determined by RT-qPCR and western blot analysis. *p < 0.05 vs. the mimic NC group. Statistical values were measured and are expressed as the mean ± standard deviation. Comparisons between two groups were analyzed by unpaired *t*-tests.

To further explore the upstream regulatory mechanism of Kcnk2, we predicted the miRNAs targeting Kcnk2 using the miRanda (http://www.microrna.org/microrna/home.do), miRSearch (https://www.exiqon.com/miRSearch) and miRmap (https://mirmap.ezlab.org/) databases. The results showed that there were 3 possible miRNAs that could target Kcnk2, namely, miR-27a, miR-27b and miR-214 (Fig. 2a). Pearson correlation analysis revealed that only miR-214 was negatively correlated with Kcnk2 in the rat spinal cord tissues of the rats exposed to I/R (Fig. 2b). RT-qPCR confirmed that miR-214 expression was significantly increased in the I/R rats compared to the sham rats (*p* *<* 0.05). Similar findings were observed in the PC12 cells exposed to H/R (*p* *<* 0.05; Fig. 2c). According to the predicted binding sites between miR-214 and Kcnk2 in PC12 cells (Fig. 2d), the luciferase activity of WT-Kcnk2-3′-UTR and MUT-Kcnk2-3′-UTR in the PC12 cells with cotransfection of either mimic NC or miR-214 mimic was measured after H/R. The luciferase activity of WT-Kcnk2-3′-UTR in the PC12 cells transfected with the miR-214 mimic showed a notable decrease compared with that after transfection of the NC mimic (*p* < 0.05), while the luciferase activity of MUT-Kcnk2-3′-UTR cotransfected with the miR-214 mimic was not altered (*p* > 0.05; Fig. 2e). Kcnk2 expression was measured in the PC12 cells transfected with miR-214 mimic or mimic NC to investigate the effect of miR-214 on Kcnk2 expression. The results demonstrated that Kcnk2 expression was substantially decreased in response to miR-214 mimic transfection compared to mimic NC transfection in PC12 cells (*p* *<* 0.05; Fig. 2f). The aforementioned findings suggest that miR-214 could target and inhibit the expression of Kcnk2 in PC12 cells.

### The transcription factor Foxd3 promotes transcriptional regulation of miR-214

**Fig. 3:**
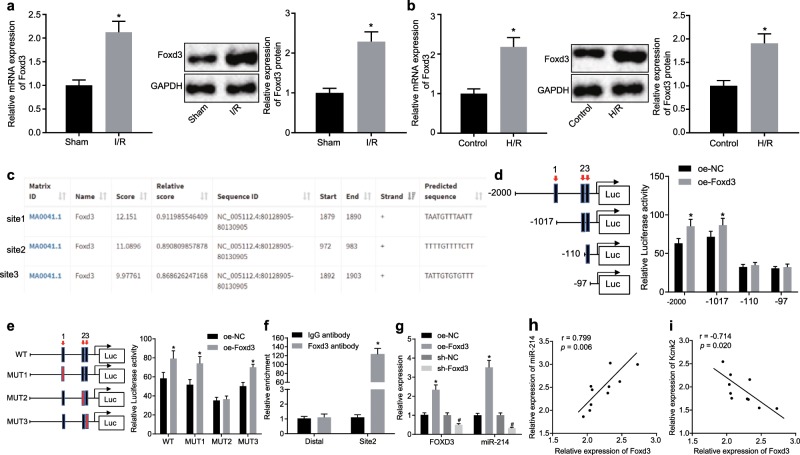
Foxd3 increases the expression of miR-214. **a** The expression of Foxd3 in the rat spinal cord tissues following IR was detected by RT-qPCR and western blot analysis. *p < 0.05 vs. the sham group. **b** Foxd3 expression in the PC12 cells exposed to H/R was determined by RT-qPCR and western blot analysis. *p < 0.05 vs. the control group**. c** Three predicted binding sites of the Foxd3 protein in the miR-214 promoter region are shown. **d** Cotransfection of the recombinant truncated miR-214 promoter luciferase reporter vector and the Foxd3 expression vector in PC12 cells as verified by dual luciferase reporter gene assays. *p < 0.05 *vs*. the oe-NC group. **e** Cotransfection of the mutant miR-214 promoter recombinant vector and the Foxd3 expression vector in PC12 cells was verified by dual luciferase reporter gene assays. *p < 0.05 vs. the oe-NC group. **f** Binding of Foxd3 to the miR-214 promoters was tested by ChIP assays. *p < 0.05 vs. the IgG antibody group. **g** The expression levels of Foxd3 and miR-214 were measured by RT-qPCR. *p < 0.05 vs. the oe-NC group. #*p* < 0.05 vs. the sh-NC group. **h** The correlation of the expression levels of Foxd3 and miR-214 in the rat spinal cord tissues following IR was confirmed by Pearson correlation analysis. **i** The correlation of the expression levels of Foxd3 and Kcnk2 in the rat spinal cord tissues following IR was verified by Pearson correlation analysis. Statistical values were measured and are expressed as the mean ± standard deviation. One-way ANOVA was used for the comparisons of data among multiple groups, followed by Tukey’s post hoc tests. Comparisons between two groups were performed by unpaired *t*-tests.

Previous literature has reported that the transcription factor Foxd3 could activate miR-214 in human colon cancer cells^14^. In addition, knockdown of Foxd3 can reduce neurotoxicity in a model of intractable epilepsy generated with primary hippocampal neurons and SH-SY5Y cell lines^26^. Based on this finding, we hypothesized that the Foxd3/miR-214/Kcnk2 signaling cascade could mediate the exacerbation of SCII. First, RT-qPCR and western blot analysis were conducted to determine the expression of Foxd3 in both the rat spinal cord tissues following I/R and the H/R-conditioned PC12 cells. The results demonstrated that the expression of Foxd3 was significantly increased in both the rat spinal cord tissues following I/R (Fig. 3a) and the H/R-conditioned PC12 cells (Fig. 3b) compared to the corresponding controls (*p* *<* 0.05). Subsequently, the three strongest potential sites for Foxd3 protein binding to the promoter region of miR-214 were predicted using the UCSC (http://genome.ucsc.edu/) and JASPAR (http://jaspar.genereg.net/) websites (Fig. 3c). We constructed a recombinant luciferase reporter vector by replacing the original promoter sequence with the wild-type or mutated truncated miR-214 promoter region. A dual luciferase reporter assay was utilized to verify the specific binding sites of Foxd3 in the miR-214 sequence. The results indicated that Foxd3 specifically bound to site 2 (TTTTGTTTTCTT) in the promoter region of miR-214 (Fig. 3d, e). The binding ability of Foxd3 to the miR-214 promoter region at site 2 was further substantiated by ChIP assays of PC12 cells (Fig. 3f). After H/R treatment, the PC12 cells were transfected with oe-NC, oe-Foxd3, sh-NC and sh-Foxd3. The expression of Foxd3 and miR-214 was assessed by RT-qPCR following H/R treatment. The results demonstrated that Foxd3 positively regulated the expression of miR-214 (Fig. 3g). Next, Pearson correlation analysis confirmed that the expression levels of Foxd3 and miR-214 were positively correlated in the spinal cord tissues of rats following I/R (Fig. 3h), while the expression levels of Foxd3 and Kcnk2 were negatively correlated in the spinal cord tissues of rats following I/R (Fig. 3i). The aforementioned findings revealed that the transcription of miR-214 was promoted by the transcription factor Foxd3 in response to IR in rat spinal cord tissues.

### The Foxd3/miR-214/Kcnk2 axis is implicated in the process of SCII

**Fig. 4:**
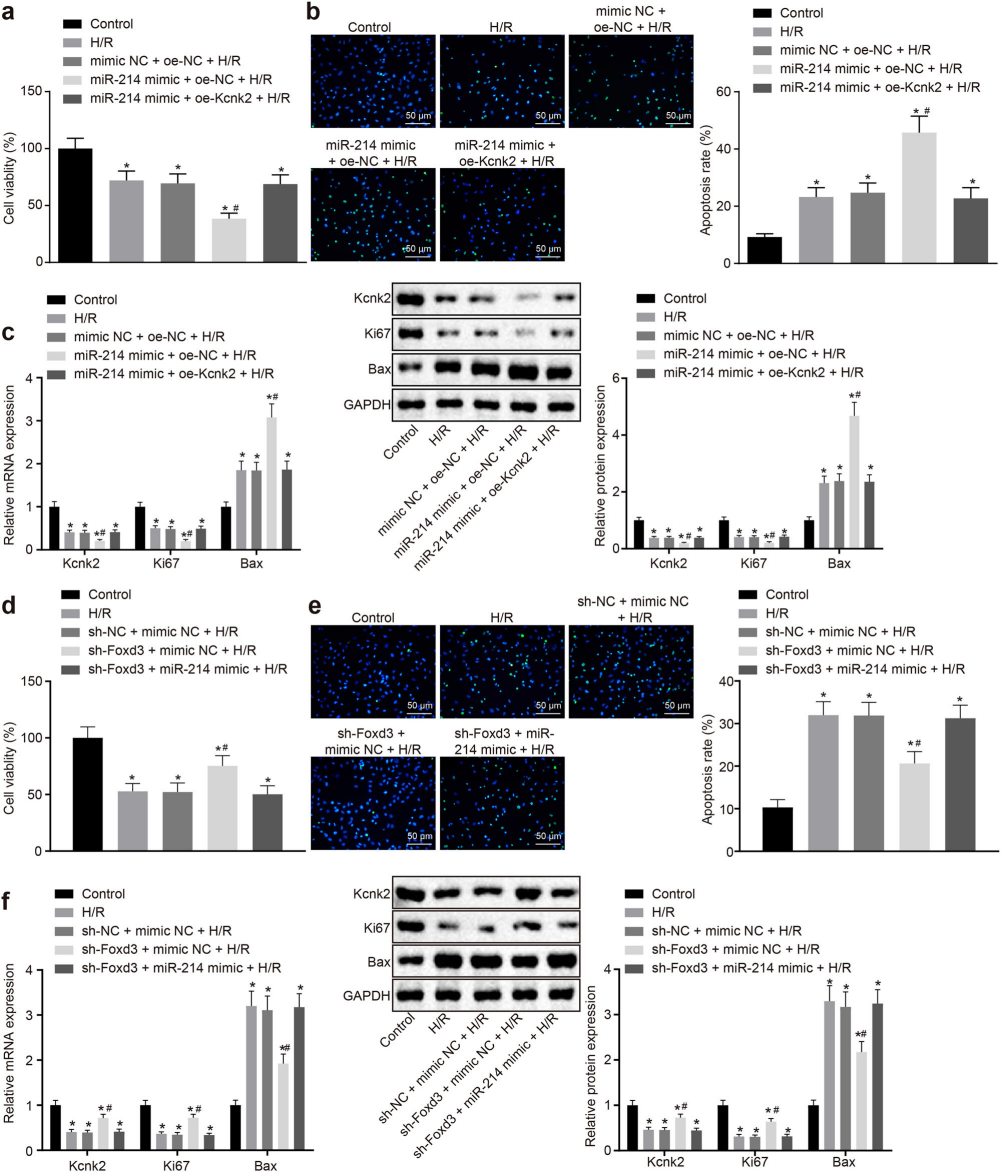
Silencing of Foxd3 impairs the miR-214-dependent inhibition of Kcnk2. **a** Cell viability was assessed by CCK-8 assays. **b** Cell apoptosis was evaluated by TUNEL assays (×200). **c** The expression levels of Kcnk2, Ki67 and Bax were detected by RT-qPCR and western blot analysis. **d** Cell viability was tested by CCK-8 assays. **e** Cell apoptosis was determined by TUNEL assays (×200). **f** The expression levels of Kcnk2, Ki67, and Bax were identified by RT-qPCR and western blot analysis. *p < 0.05 vs. the control group. #*p* < 0.05 *vs*. the H/R group, the oe-NC + H/R group, the miR-214 mimic + oe-Kcnk2 + H/R group, the sh-NC + mimic NC + H/R group or the sh-Foxd3 + miR-214 + H/R group. Statistical values were measured and are expressed as the mean ± standard deviation. One-way ANOVA was used for the comparisons of data among multiple groups, followed by Tukey’s post hoc test.

For analysis of the effects of the Foxd3/miR-214/Kcnk2 axis on SCII, PC12 cells exposed to H/R were cotransfected with the miR-214 mimic and oe-Kcnk2 and exposed to H/R. The results showed that the cell viability and Kcnk2 and Ki67 expression were significantly reduced, while the cell apoptosis and Bax expression were strongly elevated in the PC12 cells exposed to H/R compared to the control cells (*p* < 0.05). However, the effects of the H/R treatment on cell viability, apoptosis and relevant gene expression were further strengthened by transfection of the miR-214 mimic and H/R treatment. Furthermore, cotransfection of the miR-214 mimic and oe-Kcnk2 after exposure to H/R reversed the inhibitory effect of the miR-214 mimic on cell viability and the stimulatory effect on cell apoptosis (*p* < 0.05) (Fig. 4a–c).

Next, the PC12 cells exposed to H/R were cotransfected with sh-Foxd3 and/or miR-214. The cell viability of each group was measured by CCK-8 assays. The results showed that knockdown of Foxd3 in PC12 cells after H/R exposure significantly improved the cell viability (*P* < 0.05), and the rescue effect of sh-Foxd3 was abolished after miR-214 expression was restored (Fig. 4d). Cell apoptosis was then detected by TUNEL staining, and the results were consistent with those of the CCK-8 assays (Fig. 4e). Finally, RT-qPCR and western blot analysis were performed to assess the expression of Kcnk2, Ki67 and Bax. Consistent with the results above, knockdown of Foxd3 restored Kcnk2 and Ki67 expression while inhibiting Bax expression (*p* < 0.05). However, miR-214 transfection in the presence of sh-Foxd3 diminished Kcnk2 and Ki67 expression while potentiating Bax expression (Fig. 4f). Therefore, we concluded that knockdown of Foxd3 promoted the H/R-decreased Kcnk2 expression, thereby alleviating SCII.

### Knockdown of Foxd3 alleviates SCII in vivo

**Fig. 5:**
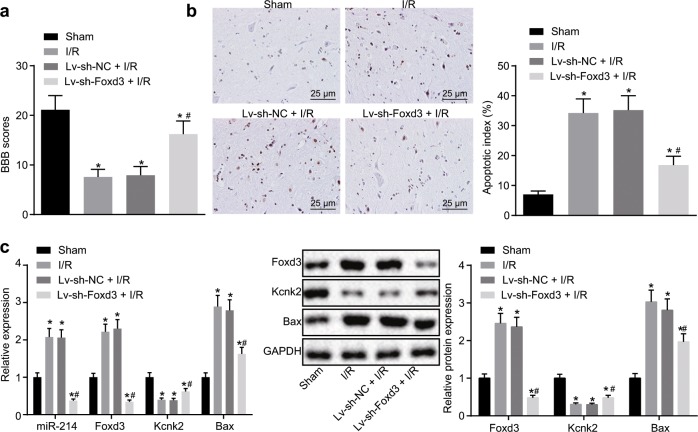
Downregulation of Foxd3 alleviates SCII in rats. **a** The locomotor function of the hind limbs was assessed by the BBB exercise rating scale. *n* = 10. **b** The apoptosis of neurons in the spinal cord tissues of rats following I/R was measured by TUNEL staining (×400). **c** The expression levels of miR-214, Foxd3, Kcnk2, and Bax in the spinal cord tissues of rats following I/R were determined by RT-qPCR and western blot analysis. *p < 0.05 vs. the sham group. #*p* < 0.05 vs. the I/R group or the Lv-sh-NC + I/R group. Statistical values were measured and are expressed as the mean ± standard deviation. One-way ANOVA was used for the comparisons of data among multiple groups, followed by Tukey’s post hoc test.

To investigate the effect of Foxd3 on SCII in rats, we injected the rats with Lv-sh-NC or Lv-sh-Foxd3 after exposure to I/R. Subsequently, the BBB locomotor rating scale was used to evaluate the hind limb motor function of the rats after 7 days of surgery. The results revealed that the locomotor activity of the rats injected with Lv-sh-Foxd3 following exposure to I/R was significantly improved, as indicated by the increased BBB scores (*p* < 0.05), given that Lv-sh-NC did not alter the BBB scores of the rats following I/R exposure (Fig. 5a). Next, the apoptosis of spinal neurons in the rat spinal cord tissues was detected using TUNEL staining. The results showed a significant decrease in the apoptosis rate of the spinal neurons in the rats exposed to I/R upon Lv-sh-Foxd3 injection (*p* < 0.05). Notably, Lv-sh-NC injection did not alter the apoptosis rate in the rats exposed to I/R (Fig. 5b). Next, the expression levels of Foxd3, miR-214, Kcnk2, and Bax in the rat spinal cord tissues were assessed by RT-qPCR and western blot analyses. The results were consistent with the above findings showing that Foxd3 knockdown mediated via Lv-sh-Foxd3 injection after exposure to I/R significantly decreased the Foxd3 and miR-214 expression (*p* < 0.05). Kcnk2 expression was restored upon Lv-sh-Foxd3 injection in the rats exposed to I/R, along with suppressed Bax expression (*p* < 0.05; Fig. 5c). These results suggested that the reduced expression of Foxd3 could ameliorate SCII in rats.

## Discussion

MiRNAs have been identified as major inflammatory regulators in SCI and other neurodegenerative diseases^27^. This study aimed to investigate the effect of Foxd3-dependent miR-214 transcription on the viability of H/R-treated PC12 cells and the motor functions of I/R-exposed rats. Our findings suggested that Foxd3 potentiates miR-214-dependent inhibition of Kcnk2 and may in turn contribute to the exacerbation of SCII.

Initially, our results showed that Kcnk2 was poorly expressed in SCII and that the overexpression of Kcnk2 enhanced the cell viability and restrained the cell apoptosis of PC12 cells. Based on previous literature, Kcnk2 plays an important role in the neuroprotection of spinal cord ischemia^25^. In addition, knockdown of Foxd3 reduced neurotoxicity in a moFcentridel of intractable epilepsy constructed by primary hippocampal neurons and SH-SY5Y cell lines^26^. The absence of Kcnk2 could impair the function of the blood-brain barrier, aggravate the inflammatory cascade and neuron apoptosis, and inhibit the recovery of nerve function after cerebral hemorrhage^28^. Kcnk2 has been reported to involve in excitatory tissues and plays an important role in other cellular mechanisms, including neuroprotection, anesthesia, and depression^29^. Kcnk2 has the potential to reduce encephalomyelitis scores and lymphocyte infiltration into the central nervous system, indicating that drugs targeting the regulation of Kcnk2 activity may be used to treat various BBB injury-related neurological diseases^30^.

Interestingly, upregulated levels of miR-214 have been reported during the development of heart failure and ischemic injury^31^. The upregulation of miR-214 expression was also observed following I/R exposure^32^. Bax, a member of the Bcl-2 family of cell death mediators, was shown to be a vital promoter of programmed cell death^33^. Upregulation of miR-214 reduced the overexpression of Nav1.3 and Bax after SCI generated by electroacupuncture^34^. Moreover, in injured adult rat spinal cord, a concomitant elevation of proliferation, as shown by the mitotic markers Ki67 and bromodeoxyuridine, was found^35^. A miR-214 inhibitor decreased the apoptotic index of hepatic cells and increased the Ki67 positivity in bone marrow-derived mesenchymal stem cells following transplantation^36^. Furthermore, miR-214 could target Kcnk2, as shown by dual luciferase reporter gene assays. The upregulation of ALCAM mediated by miR-214 plays a key role in the metastasis and proliferation of cancer cells^37^. Wang et al. showed that the overexpression of miR-214 could promote the cell invasion of breast cancer through the regulation of p53 expression, thereby facilitating the progression of breast cancer^38^.

In addition, the expression levels of Foxd3 and miR-214 were positively correlated in rat spinal cord tissues following I/R, as Foxd3 could activate miR-214. A prior study has shown that in colorectal cancer, Foxd3 regulates miR-214 transcription, thereby restraining metastasis and invasion^14^. Our western blot and RT-qPCR results showed upregulated levels of Foxd3 in the rat spinal cord tissues following I/R. Foxd3 is a transcription factor that has been shown to be necessary for early neural crest-derived progenitor self-renewal, pluripotency and establishment of various neural crest-derived cells and structures, including the intestinal nervous system^39^. Foxd3 can also suppress melanogenesis in the neural crest and was found to be downregulated due to melanoblast migration^40^.

**Fig. 6:**
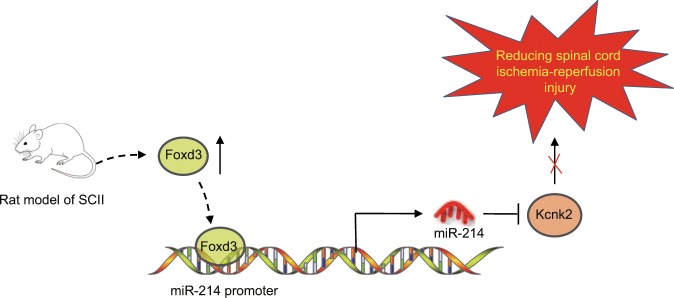
Regulatory mechanism of the Foxd3/miR-214/Kcnk2 axis in the progression of SCII. Foxd3 expression was significantly increased in the SCII rats. Foxd3 can also potentiate the transcriptional regulation of miR-214 on Kcnk2, thereby inhibiting the expression of Kcnk2 and contributing to the exacerbation of SCII.

In conclusion, the key findings from this study suggest that Foxd3 potentiates miR-214-dependent Kcnk2 inhibition, contributing to the exacerbation of SCII (Fig. 6). Therefore, the identification of the Foxd3/miR-214/Kcnk2 axis might provide further insight into the underlying mechanisms of SCII. Furthermore, either Foxd3 or miR-214 may potentially serve as a therapeutic target for the treatment of SCII in the future. However, the specific molecular mechanisms of the Foxd3/miR-214/Kcnk2 axis in SCII require further investigation.
